# Use of pulse field ablation in the treatment of an atrial tachycardia: A case report

**DOI:** 10.1002/ccr3.9058

**Published:** 2024-06-11

**Authors:** Noha Elbanhawy

**Affiliations:** ^1^ Cardiology Department, Castle Hill Hospital Hull University Teaching Hospitals Hull UK

**Keywords:** atrial tachycardia, electroanatomic mapping, pulmonary veins, pulse field ablation

## Abstract

This case illustrates the safety and efficacy of pulse field ablation (PFA) in the short and medium term. It could be used to isolate extra pulmonary sites including the posterior wall. PFA could be used in the treatment of atrial tachycardias both focal and reentrant in combination with ultrahigh density electroanatomic mapping. It is also important to understand that different catheter shapes are available and their use can be tailored to the targeted anatomical site.

## Graphical abstract

Figure illustrating atrial flutter on ECG (A), intracardiac (B) and termination to sinus rhythm (C).

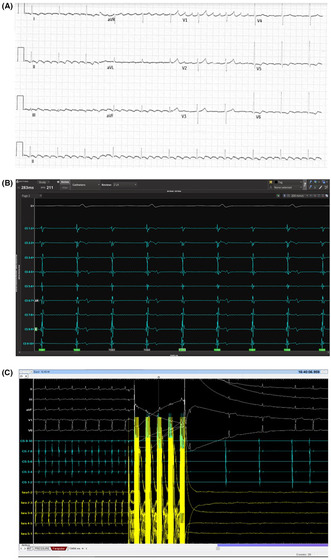

## 1 INTRODUCTION

Atrial flutter is the second most common arrhythmia after atrial fibrillation. Incidence and prevalence are less well known, however. It is a macroreentrant circuit that usually spans a large area of the atria. It usually develops as a result of the presence of a fixed barrier whether anatomical, for example, valves and scar, or simply a functional barrier due to changes in conduction velocity and recovery of cardiac myocytes which helps initiate and maintain the circuit. Common flutter is the counterclockwise peritricuspid flutter (cavotricuspid isthmus‐dependent CTI) but other types of flutter that sometimes overlap in features also exist. These could be right or left atrial flutters. Commonly atrial flutter is difficult to treat especially with rate control medications and restoration and maintenance of sinus rhythm often ultimately requires an ablation procedure as in the following case.

Pulse field ablation (PFA) is a new technology that has been approved for the isolation of pulmonary veins in paroxysmal and persistent atrial fibrillation. Using PFA for targeting extra pulmonary sites combined with ultrahigh density 3D electroanatomic mapping (EAM) is an innovative approach that has been reported as single‐center experiences. Our case is an example of such an approach where PFA was used to acutely terminate a macro reentrant scar‐related left atrial flutter and isolate the posterior wall.

## 2 CASE HISTORY

A 54‐year‐old male with history of previous mitral valve repair (2019) and paroxysms of atrial fibrillation was admitted to hospital with palpitations and a picture of heart failure.

Examination on admission showed raised neck veins, bilateral basal crepitations and pitting edema up to mid shins, all suggestive of a subacute or chronic process. He was peripherally perfused and observations were stable with systolic blood pressure ranging between 100 and 120 mmHg. His heart rate initially was uncontrolled and ranging between 140 and 150 bpm.

A transthoracic echocardiogram showed severe left ventricular systolic dysfunction with dilated atria and well‐functioning mitral valve showing mild mitral regurgitation. Pulmonary artery systolic pressure was raised and estimated at 40 mmHg. The patient was initially treated with conservative antifailure measures including furosemide infusion, fluid restriction, and standard heart failure drugs and the heart rate was better controlled with uptitration of his beta blocker therapy. It was concluded that the most likely cause of failure was his persistent flutter.

**FIGURE 1:**
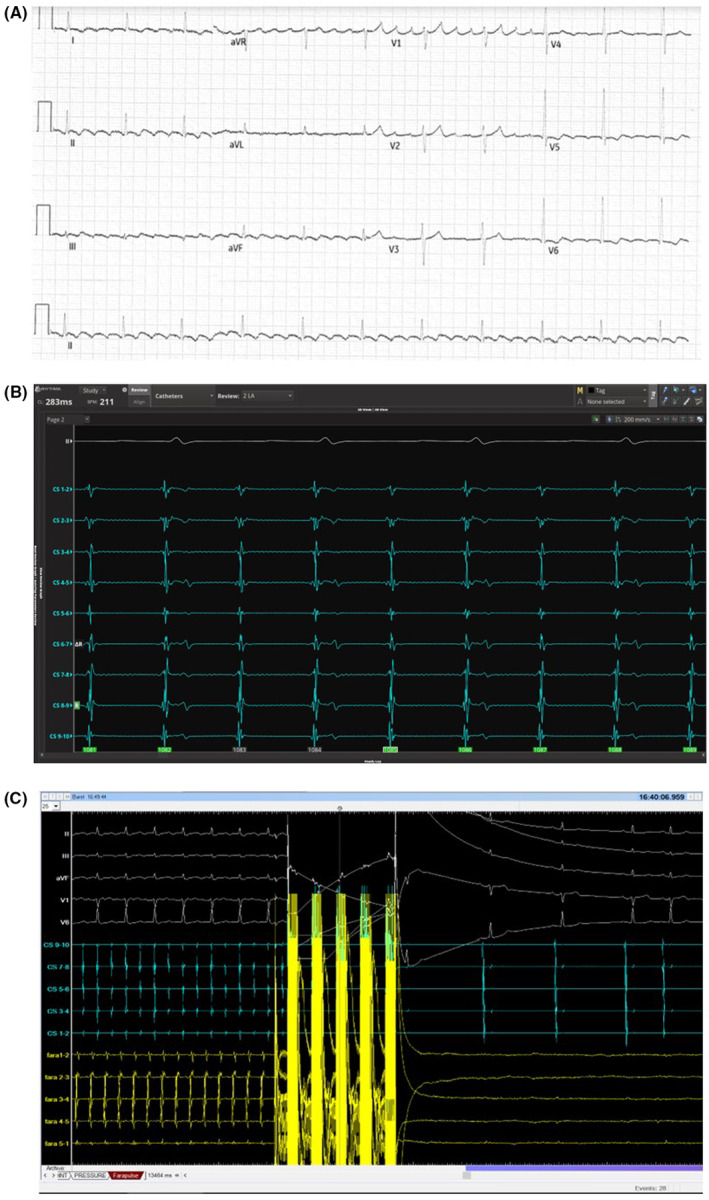
(A) Twelve‐lead ECG during tachycardia. Note typical counterclockwise flutter cannot be excluded. (B) Intracardiac recording of coronary sinus activation. Note proximal to distal activation. (C) Intracardiac recording showing coronary sinus activation (blue) and Farawave catheter signals in right lower pulmonary vein (RLPV) (yellow) with termination of tachycardia. Note loss of pulmonary vein potentials on Farawave bipolar electrograms post lesion delivery.

The ECG features were suggestive of a counterclockwise typical flutter but a left‐sided tachycardia could not be ruled out (Figure 1A).

## 3 METHODS

**FIGURE 2:**
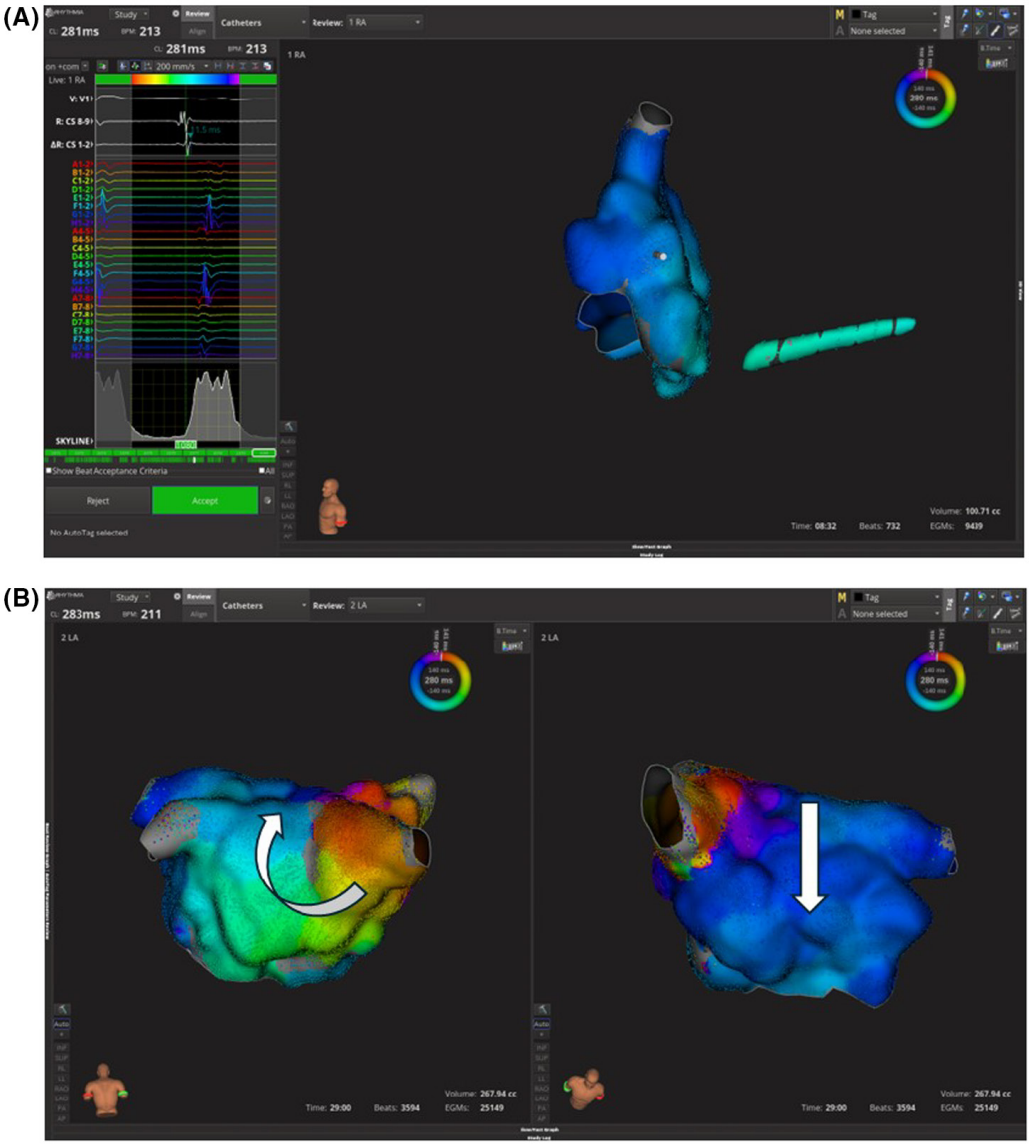
(A) Electroanatomic map showing passive activation of right atrium. Note skyline showing large missing portion of tachycardia cycle length. (B) Macro reentrant circuit propagating around scar on left atrium (LA) posterior wall (left image). Passive activation of LA anterior wall (right image). *Note*: Arrows showing direction of propagation of wavefront.

**FIGURE 3:**
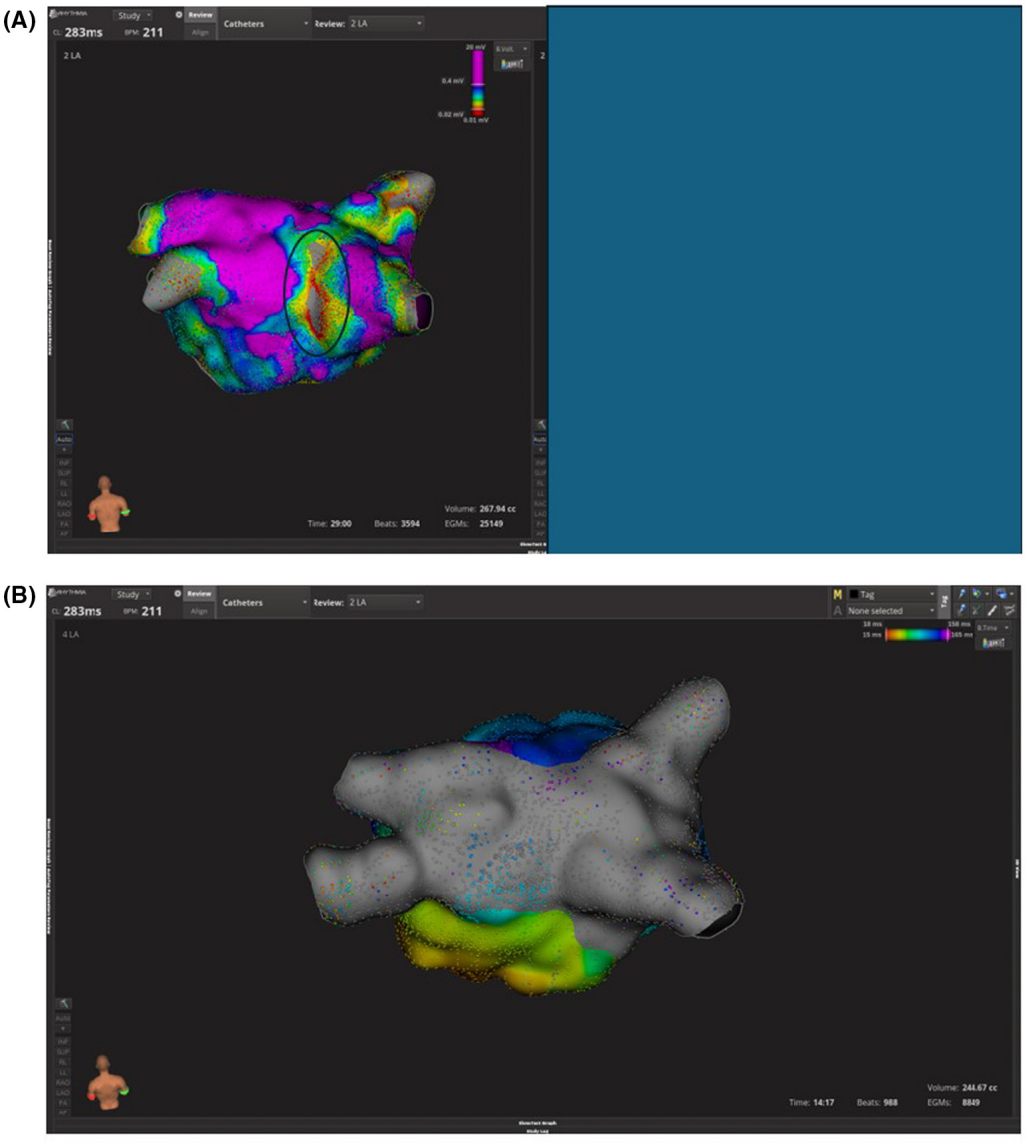
(A) Left atrium (LA) map pre‐ablation showing low voltage of posterior wall with area of scar (gray highlighted area). (B) LA map post pulse field ablation (PFA). Note silent posterior wall. Scale of bipolar voltage maps is 0.02–0.4 mv.

The patient was brought to the EP lab. Coronary sinus activation was proximal to distal with a slight chevron‐like appearance (Figure 1B). Mapping of the left atrium (LA) with Rhythmia system (Boston Scientific, Marlborough, MA) using INTELLA MAP ORION™ catheter showed passive activation of the right atrium and absence of a large portion of the tachycardia cycle length (Figure 2A). Mapping of the LA showed a macro‐reentrant flutter around a posterior wall scar close to the right lower pulmonary vein (RLPV) with passive activation of the anterior wall (Figure 2B, Video 1). FARAPULSE PFA™ System (Boston Scientific, Marlborough, Massachusetts) was used to isolate the pulmonary veins (PVs). Termination of the tachycardia during RLPV ablation was observed (Figure 1C). Posterior wall ablation was then attempted with PFA to encompass the arrhythmia substrate. Remapping showed silent PVs and posterior wall compared to the initial voltage map (Figure 3A,B).

**VIDEO 1:** Video showing a PA view of the left atrium with propagation of the re‐entrant tachycardia wavefront around a scar close to the right inferior pulmonary vein. Note the whole tachycardia cycle length is seen as depicted by the colour scheme (red to violet).

## 4 DISCUSSION

Ablation as a method of treating supraventricular arrhythmias especially preexcitation syndromes initially developed in 1969. As success was achieved, eventually the indications expanded including other arrhythmias such as atrial flutter and ventricular tachycardias. The aim of ablation was to achieve transmural contiguous lesions that destroyed arrhythmogenic tissue. Initially direct current (DC) was used but because of issues such as barotrauma and recurrence of arrhythmias, alternative sources of energy were studied and eventually used. This led to the use of thermal energy such as radiofrequency (RF) and cryoablation (CA) both effective but associated with injury to collateral structures causing infrequent but serious complications such as atrioesophageal fistulae, pulmonary vein stenosis, and phrenic nerve palsy. This led to the resurgence of DC in an effort to find safer sources of energy. Several preclinical studies were conducted using DC delivered as pulses and creating a local electrical field affecting the cell lip bilayer and forming nanopores with variable effects some of which were irreversible and leading to Adenosine triphosphate (ATP) exhaustion, ion channel failure, calcium overload, and loss of cell homeostasis leading to cell death. It was found to be effective but also provided safety due to tissue selectivity and it seemed that cardiac tissues were more vulnerable to its effects.^1^, ^2^

PFA as a novel source of energy thus has become increasingly used in multiple centers throughout Europe and North America. Because of its tissue selectivity, it thus has the potential of sparing extracardiac structures, which has always been a concern when performing AF ablations.

It has proven its safety and efficacy in the short and medium term in a number of single and multicenter studies. Acute success has been reported to be close to 100% and it has proven non‐inferiority to radiofrequency ablation in terms of freedom from symptomatic AF at 12 months. Some studies also proved durability of lesions with remapping at 90 days post initial procedure.^3^, ^4^

In a recent meta‐analysis comparing PFA to other thermal energy sources, that is, RF and CA, 24 landmark studies were compared in terms of safety (perioprocedural complications) and efficacy (total procedure times/LA dwell times) and freedom for atrial arrhythmias at 1 year. It concluded that there were significantly lower complications with PFA compared to other thermal ablations, PFA group (2.05%; 95% CI 0.94–3.46) compared to the thermal ablation group (7.75%; 95% CI 5.40–10.47) (*p* 0.001). There was also no significant difference in recurrence of atrial arrhythmias, PFA group (14.24%; 95% CI 6.97–23.35) compared to the thermal ablation (25.98%; 95% CI 15.75–37.68) (*p* = 0.132). There was also no difference in procedure and fluoroscopy times though LA dwell times were shorter (less than 1 h) in the PFA groups.^2^

Recently, a randomized trial comparing PFA to RF and CA. It was a single‐blind non‐inferiority study where paroxysmal AF patients were assigned in a 1:1 fashion to PFA (*n* = 305) compared to RF/CA (*n* = 302). PFA proved to be non‐inferior to the other group with respect to freedom from a composite of initial procedural failure, documented atrial tachyarrhythmia after a 3‐month blanking period, antiarrhythmic drug use, cardioversion, or repeat ablation and with respect to device‐ and procedure‐related acute and chronic serious adverse events at 1 year.^5^

Although in vivo studies have shown that contact force is not essential to create lesions using PFA compared to RFA, a certain degree of contact is essential to create deep and durable lesions.

Thus one of the limitations and areas requiring further study is transmurality of lesions and long‐term durability. The effect of contact force and the interplay with PFA pulses was recently studied on 11 swine models in the ventricular myocardium. It was found to be essential and synergistic in creating lesions though it was concluded that further studies would be needed to create an index with the optimal PFA dose and contact force for transmural lesions in the atria and ventricles.^6^

Different catheters have become available thus allowing the targeting of various sites including the posterior wall that was reported in recent studies. Tissue selectivity has allowed delivering lesions safely and effectively with PFA without causing significant collateral damage.^7^, ^8^

The use of PFA in the treatment of focal and reentrant left ATs has also been reported in isolated case reports^9^ and as single‐center experiences with a limited number of patients.^10^, ^11^, ^12^ In those studies, PFA was combined with EAM.

There was acute termination of all the tachycardias although focal catheters seemed to be more suited for ablation in those cases especially with anterior and mitral isthmus lines.^11^

In this case, we used what was learned from previous experience. We used EAM to show the reentrant circuit and voltage of the LA and combined it with PFA. We then proceeded to isolate the veins, terminate the tachycardia, and also perform a posterior wall ablation. It is yet to be seen if this will translate into future long‐term freedom from atrial arrhythmias.

## 5 CONCLUSION

PFA could successfully be used to deliver lesions beyond the pulmonary veins with successful acute termination of organized atrial tachycardias (ATs), in this case a scar‐related macro reentrant circuit. No acute complications were observed.

## AUTHOR CONTRIBUTIONS

**Noha Elbanhawy:** Writing – original draft; writing – review and editing.

## FUNDING INFORMATION

The author did not receive financial support for the research, authorship, and/or publication of this article.

## CONFLICT OF INTEREST STATEMENT

There are no conflicts of interest to declare.

## CONSENT

Written informed consent was obtained from the patient to publish this report in accordance with the journal's patient consent policy.

## Data Availability

The data that support the findings of this study are available from the corresponding author upon reasonable request.
